# Semantic congruent audiovisual integration during the encoding stage of working memory: an ERP and sLORETA study

**DOI:** 10.1038/s41598-017-05471-1

**Published:** 2017-07-11

**Authors:** Yuanjun Xie, Yuanyuan Xu, Chen Bian, Min Li

**Affiliations:** The department of military psychology, College of Psychology, the Third Military Medical University, Chongqing, +86023730030 China

## Abstract

Although multisensory integration is an inherent component of functional brain organization, multisensory integration during working memory (WM) has attracted little attention. The present study investigated the neural properties underlying the multisensory integration of WM by comparing semantically related bimodal stimulus presentations with unimodal stimulus presentations and analysing the results using the standardized low-resolution brain electromagnetic tomography (sLORETA) source location approach. The results showed that the memory retrieval reaction times during congruent audiovisual conditions were faster than those during unisensory conditions. Moreover, our findings indicated that the event-related potential (ERP) for simultaneous audiovisual stimuli differed from the ERP for the sum of unisensory constituents during the encoding stage and occurred within a 236–530 ms timeframe over the frontal and parietal-occipital electrodes. The sLORETA images revealed a distributed network of brain areas that participate in the multisensory integration of WM. These results suggested that information inputs from different WM subsystems yielded nonlinear multisensory interactions and became integrated during the encoding stage. The multicomponent model of WM indicates that the central executive could play a critical role in the integration of information from different slave systems.

## Introduction

The integrated information derived from different sensory modalities is indispensable for providing unified perceptions^1^. Multisensory integration is the process by which information from different sensory systems is combined by nervous system units^2^, and it considerably improves accuracy^3^, reduces reaction times^4, 5^, and enhances precision^6–8^. The enhancement in behavioural performance in response to multisensory inputs lies in the convergence of information rather than in the simple redundancy of targets^9^ because multisensory stimuli increase the firing rates of cells to a level that exceeds the rate predicted by the summation of responses to unisensory stimuli^10, 11^.

Certain basic principles underlie the integration effect^12, 13^. Temporally and spatially aligned sensory inputs in different modalities contribute to further processing and result in behavioural facilitations^14–16^, and higher level factors, such as semantics or semantic congruence, are capable of influencing the integration of information across sensory modalities. In particular, semantic congruence refers to combinations of auditory and visual stimuli that are presented in terms of their meaning^17^. By assessing the consequences of presenting matching (the image and sound belong to the same object) or mismatching (the image and sound belong to different objects) information, judgements on the congruence or incongruence of stimuli pairings can be made. The impact of semantics on multisensory integration has been investigated in several studies^18, 19^, and significantly faster reaction times were found for semantically congruent audiovisual pairings compared with unisensory stimulation and significantly longer response times were found for semantically mismatched conditions. Thus, semantic congruence is a critical factor for determining multisensory behavioural performance^18^.

Additionally, the neocortex has been found to be necessary for multisensory integration to increase the efficiency of the underlying processes. Several higher-order association cortical areas were considered multisensory, including the intraparietal complex, the superior temporal sulcus, and the frontal cortex^20–23^. Intracranial recoding and neuroimaging studies in humans demonstrated that multisensory inputs were indeed co-localized to regions of the parietal lobe^22, 24–26^, including the inferior parietal sulcus, the inferior parietal lobule, and the superior parietal lobule.

Previous studies have demonstrated the benefits of multisensory perception and characterized the properties of multisensory integration by manipulating spatial, temporal and semantic signals from different modalities. However, the impact of multisensory integration on learning and memory have received limited attention^27^. Recently, the contribution of cross-modal information to perceptual learning has been studied, and subjects trained with congruent audiovisual stimuli showed an advantage when learning visual tasks compared with subjects trained only with visual stimuli^28, 29^. In addition, researchers have investigated the effect of multisensory experiences on subsequent unisensory memory performance with a continuous recognition task^3, 30–32^, and they found that recognition memory for repeated pictures was improved when the picture was initially presented with semantically congruent sounds compared to when it was presented with semantically inconsistent sounds. Similarly, subsequent studies on the relationship between sounds and recognition tasks showed that improved recognition occurred after an initially congruent picture-sound pairings^33–35^.

Electrophysiological studies from a group of researchers demonstrated an incidental effect of multisensory memory on subsequent visual stimuli processing, and they showed that the rapid differentiation of repeated visual stimuli occurred according to the subjects’ multisensory (auditory-visual) or unisensory (visual only) experiences approximately 60–136 ms post-stimulus onset^30^. The effect was observed even when initial multisensory experiences consisted of images and meaningless sounds^32^. Regions within the lateral-occipital complex were more active in response to visual stimuli associated with multisensory rather than unisensory experiences^31^. These results suggested that multisensory interactions continued to affect subsequent memory recognition^36^.

Furthermore, several studies in humans and primates indicated the presence of multisensory facilitation in working memory (WM). Heikkilä and colleagues^37^ examined the effect of audiovisual encoding in WM on later unisensory memory recognition. The participants were instructed to memorize auditory or visual stimuli that co-occurred with either a congruent stimulus, an incongruent stimulus or a neutral stimulus combinations during the encoding stage. Subsequent memory performance was improved overall when the sound stimulus was initially paired with a semantically congruent picture than a neutral stimulus. In addition, a primate behaviour study^38^ trained monkeys to learn auditory stimuli and similar visual stimuli in a delay matching-to-sample task and then tested the animals with a concurrent audiovisual matching task with equal proportions of auditory, visual, and audiovisual stimuli. Consistent with outcomes in human studies, the study found that the accuracy was higher and response times were faster for audiovisual test stimuli than those for either of the unisensory test stimuli.

Although the above findings indicate that multisensory objects processing impacts later memory performance and brain activity, the mechanisms underlying multisensory processes are not unambiguous. For multisensory experiences in WM, the influence of encoding information from different subsystems on subsequent memory performance is poorly understood and whether the information can be integrated during the encoding stage remains unclear.

The purpose of the present study was to investigate the integration effect of multisensory WM via an event-related potential (ERP) approach, and the addition criterion was applied to examine the integration effect. ERPs in response to a singular auditory stimulus (A) and visual stimulus (V) were summed and compared with the ERPs in response to simultaneously presented audiovisual stimuli (AV). If the neural response to AV differed with the sum of A and V (ERP_AV_ ≠ERP_A_ + ERP_V_), then multisensory integration was thought to have occurred. This approach has been repeatedly applied in multisensory research on ERPs in humans^39–41^. We predicted that multisensory integration would be formed in the encoding stage of WM and influence later memory recognition. Finally, cortical sources of the integration effect in multisensory WM were modelled by the standardized low-resolution brain electromagnetic tomography software (sLORETA)^42^.

## Results

### Behavioural results

**Figure 1:**
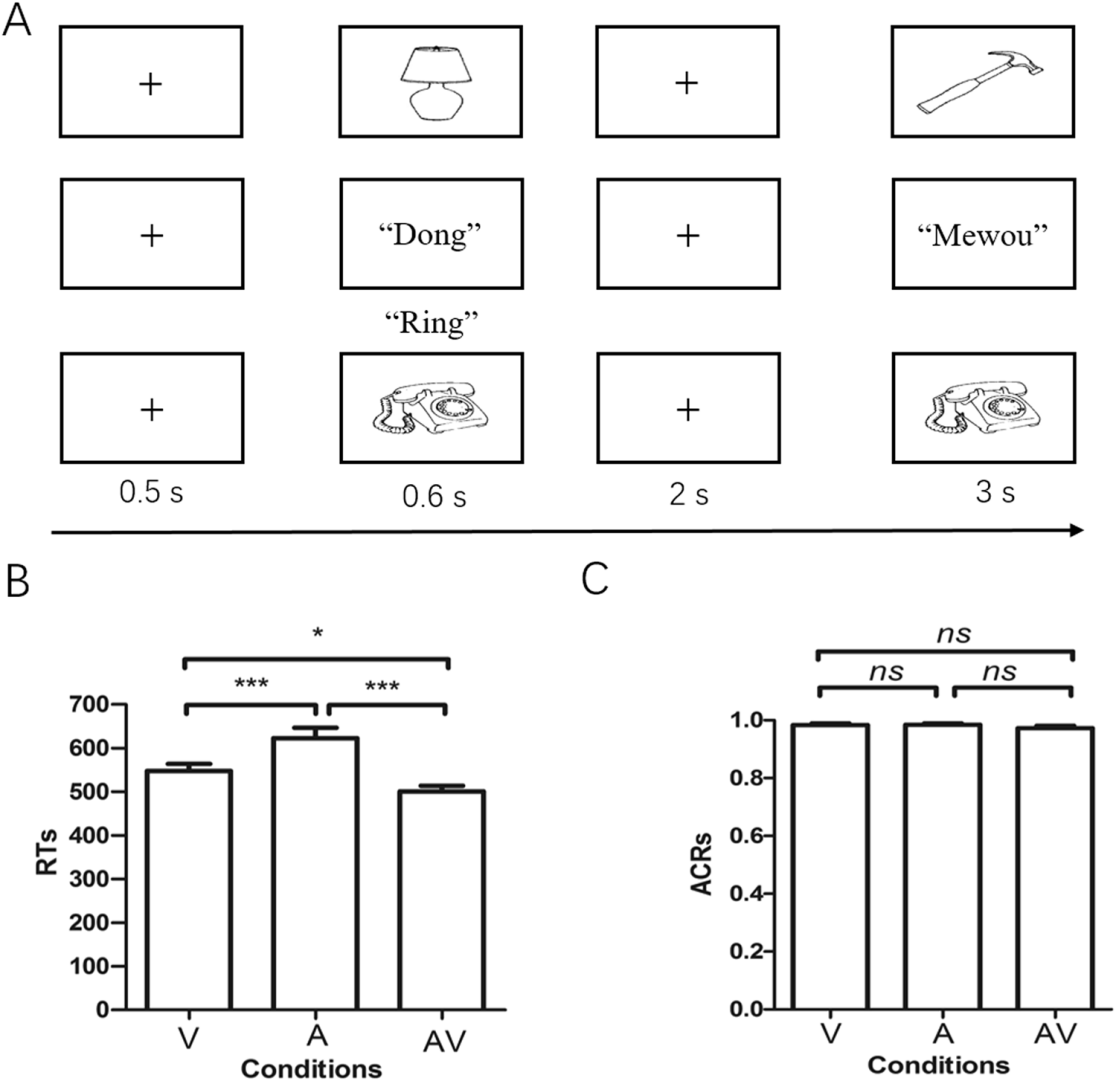
Behavioural data results. (**A**) Three-block design of the behavioural experiment. In each trial with three blocks, a fixation cross was shown for 0.5 s, and then a stimulus (visual, auditory, and congruent audiovisual) was presented, a blank screen was shown after a 2 s delay, and finally the test stimulus appeared within a 3 s time limit. (**B**) Corrected mean RTs for memory retrieval during the three conditions. (**C**) Mean ACRs for memory recognition in the three blocks. Error bars represent the standard error of the mean. Note: *p < 0.05, ***p < 0.001. *ns*, no significant difference.

A significant main effect of the modalities was observed on the mean reaction times (RTs) [F_2, 35_ = 24.31, p* < 0.001, η* ^*2*^ *= 0.42*]. Post hoc comparisons revealed that the RTs for memory retrieval under the AV condition were faster than those under the unimodal conditions (A and V). However, the accurate response rate (ACRs) for memory recognition reached a ceiling in all trial types, and significant effects were not observed during these conditions [F_2, 35_ = 2.13, p* > 0.05, η* ^*2*^ *= 0.07*]. The details of the behavioural analysis are shown in Fig. 1B and C.

### ERP results

**Figure 2:**
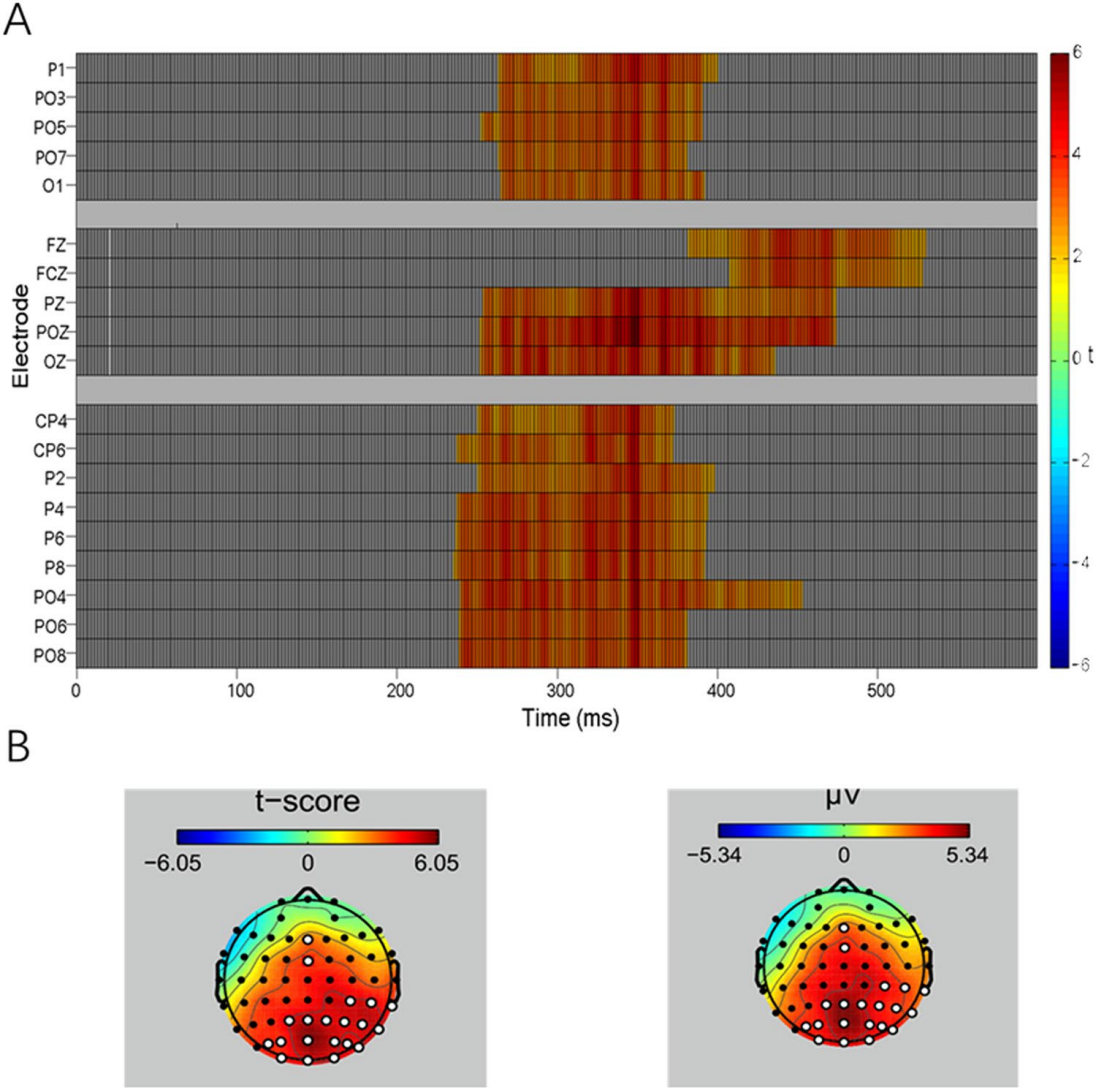
Point-wise cluster test of the integration effect with MUET. (**A**) Significant clusters in time points and electrodes (only significantly different electrodes are displayed) that occurred in the time window of approximately 236–530 ms. (**B**) Distributions of significant electrodes in the scalp topography (white dash).

**Figure 3:**
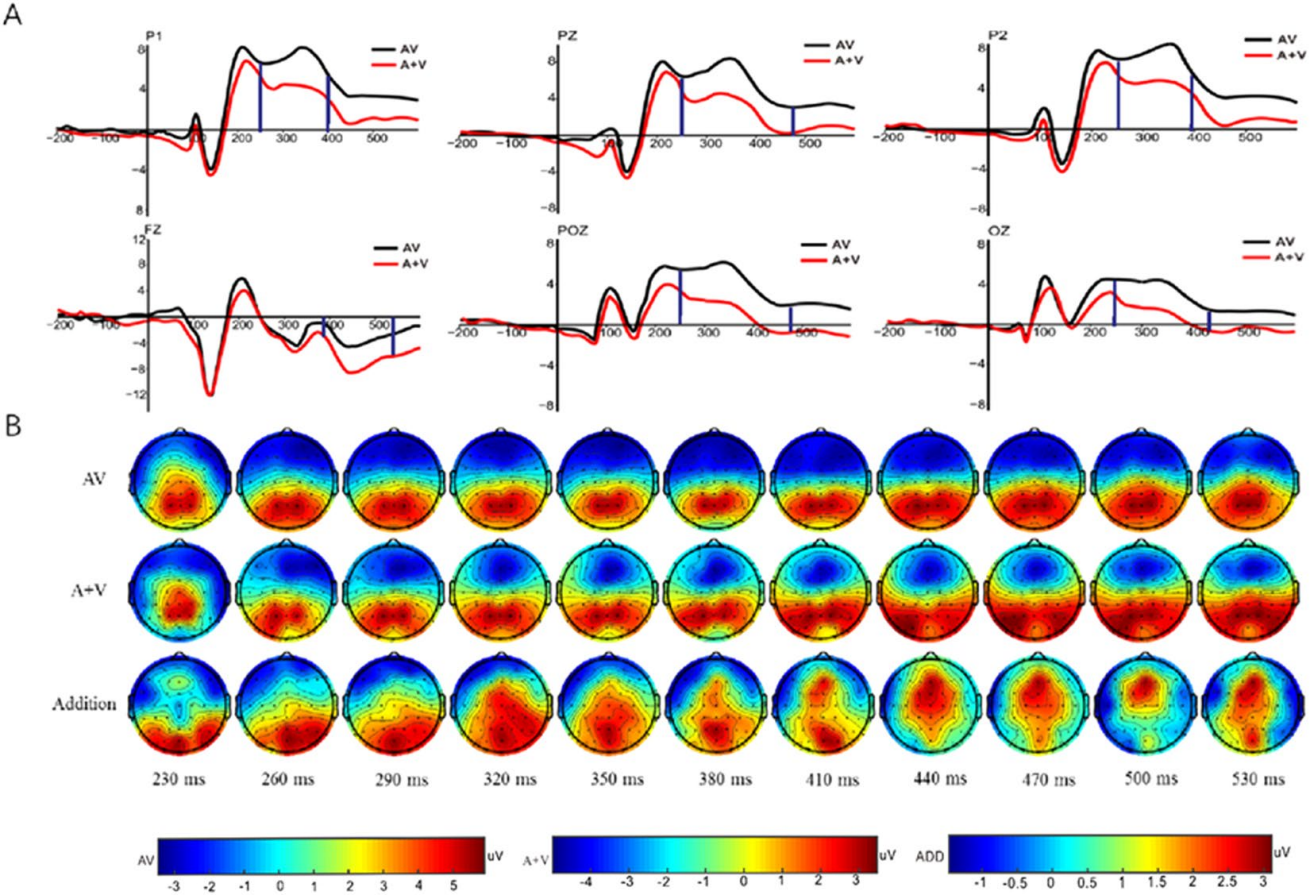
(**A**) Grand average ERP waveforms under the AV and A + V conditions at the six primary electrodes (P1, PZ, P2, FZ, POZ, and OZ); substantial differences were observed between the AV and A + V conditions for these electrodes over a latent period of 236–530 ms (between the two vertical blue lines). (**B**) Scalp topography distributions for the steps of instantaneous amplitude values during the AV, A + V, and addition[ADD, AV−(A + V)] conditions.

The difference in potential﻿s between ERP_AV_ and ERP_A+V_ via Mass Univariate ERP Toolbox (MUET) was examined to determine the emergence of the integration effect during the multisensory encoding stage of WM. The smallest significant t-score was 2.03, which corresponded to a test-wise alpha level of 0.05. The results showed that a super-additiona﻿lity effect (ERP_AV_ > ERP_A+V_) of multisensory information occurred during the encoding stage of WM within 236–530 ms after stimulus onset over the parieto-occipital electrodes and the frontal electrodes (beginning at 392 ms) (Fig. 2). The overall average ERP waveforms at six primary electrodes under AV and A + V conditions are displayed in Fig. 3. The mean amplitudes of specific electrodes significantly differed between the AV and A + V conditions over a latency period of 236–530 ms (Fz, 392–530 ms; the smallest F_1, 35_ = 10.82, p < 0.01, *η* ^*2*^ *= 2.24*). The scalp topography distributions of the three conditions (AV, A + V, and Addition) showed that audiovisual interactions primarily activated the posterior brain regions, with the frontal areas recruited in the processes at a relatively later stage.

### sLORETA results

**Figure 4:**
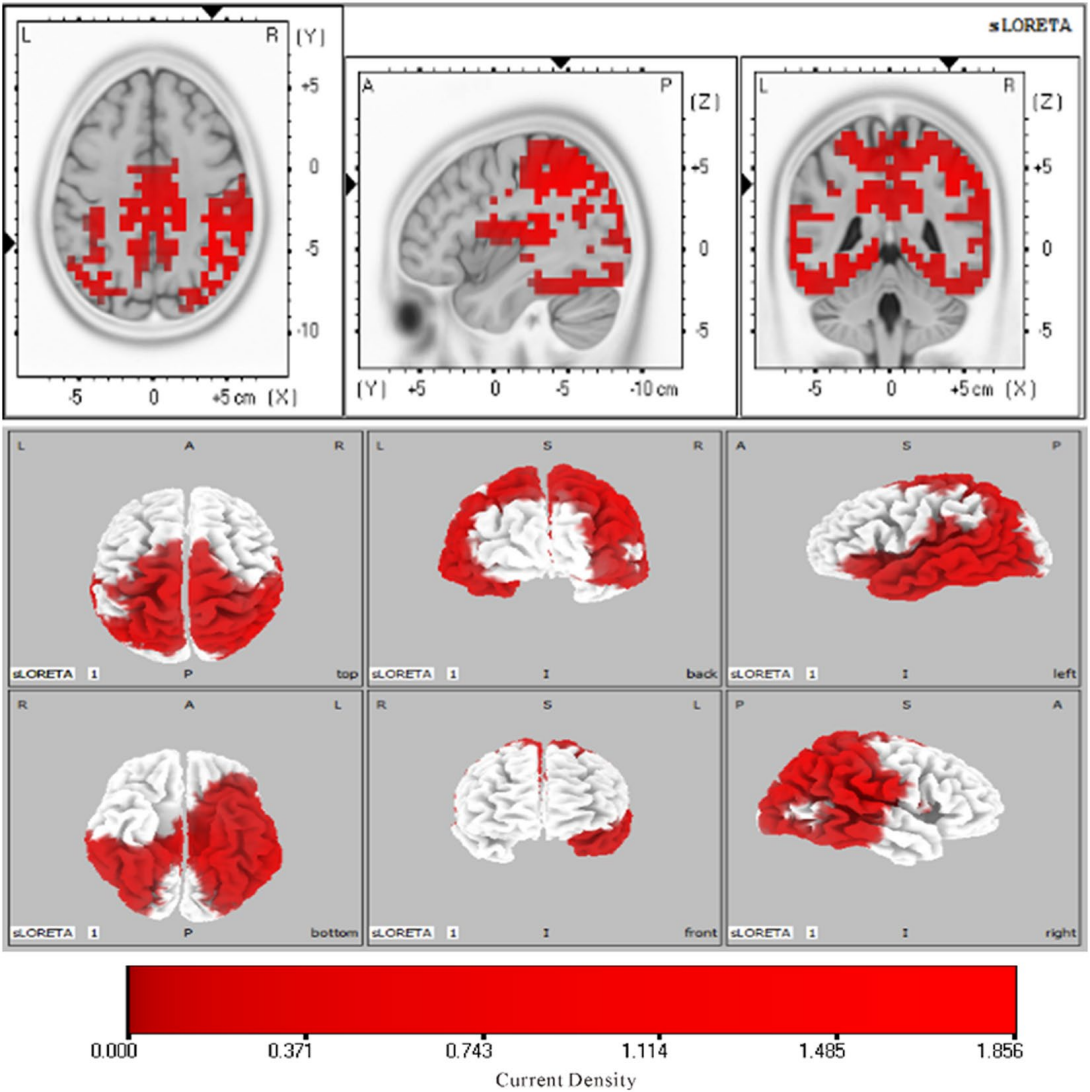
sLORETA images for the integration effect of multisensory WM over a timeframe of 236–530 ms (slice view and 3D view). The highest level of activation was localized in the inferior parietal lobule (BA 39/40).

Figure 4 showed the sLORETA brain maps that represent neural generators of scalp voltages within the temporal window (236–530 ms) of the significant integration effect. Several brain areas were revealed, including the frontal (inferior frontal gyrus, BA47; middle frontal gyrus, BA9), parietal (inferior parietal lobule, BA39/BA40; precuneus, BA7/BA31), temporal (middle temporal gyrus, BA21; superior temporal gyrus, BA41), and occipital (middle occipital gyrus, BA19) areas. The maximum sources were found in the inferior parietal lobule.

## Discussion

The present study investigated the integration effect of multisensory WM via an ERP analysis and sLORETA source location method. Behaviourally, the mean RTs for memory recognition during the semantically congruent audiovisual trial type were faster than the mean RTs of both unisensory trial types. Moreover, the ERP for AV stimuli observed in the encoding stage of WM differed from the sum of the ERPs for the A and V stimuli and appeared within 236–530 ms after stimuli onset. The typical outcome of multisensory integration is behavioural facilitation and differences in brain activity between multisensory stimuli and the summed unisensory signals^43^. The results of the present study were consistent with this proposition and suggested that the multisensory representation of WM was established during the encoding stage and later to facilitate memory retrieval. The neural origins of the integration effect in the encoding stage of WM involve distributed brain networks. This finding is consistent with the results of previous multisensory research which multiple distributed networks were implicated in multisensory integration^44^ and support the opinion that WM processing involves distributed neuronal networks^45^.

The organization of information within WM must be discussed ﻿in order to completely understand WM processing from a multisensory perspective^46^. The feature binding of WM is an important issue that indicates whether each feature of an object is arranged separately. Multiple studies have shown that the processing of colour and shape features or an object’s identity and location is automatically connected^47–50^. Other studies have demonstrated that the binding of features can even occur across sensory modalities^51, 52^. The hippocampus also appears to play an important role in the process of binding^53^ (but see ref. 54). To determine how information from different subsystems can be integrated based on mutual basic codes, the episodic buffer has been proposed as a new component of WM^55^, and it combines information presented in the different storage systems into a complex representation that depends on executive resources. However, further investigation showed that the episodic buffer is a passive domain-general buffer for the storage of information from a visuospatial sketchpad and a phonological loop according to evidence from the cross-modal binding of WM, because binding the features of colour and shape to objects across auditory and visual modalities appears to be automatic and does not require explicit attentional control^56^. Thus, although the central executive (CE) was initially considered to be a supervisory attention system, we propose that it may play a crucial role in the integration of information from different code systems. In other words, the CE involved in attention control and monitors various slave systems in a coordinated manner^57^, and it may also provide an integration function that merges the information derived from these subsystems into a temporary unitary representation. Because integration or the  capability of performing two tasks simultaneously is regarded as a necessary function of the CE^58^. For instance, Alzheimer’s patients with a defective executive function show an apparent deterioration in the coordination of the operation of two slave systems: the phonological loop and the visuospatial sketchpad^59, 60^.

The neural substrates of CE were originally assumed to be located in the frontal cortex because patients or primates with lesions in those brain regions frequently demonstrate impaired performance on a range of tasks that assess executive functions^61–63^. However, evidence also indicates that the CE component of WM is distributed between anterior and posterior brain areas^64^. Therefore, the CE not only recruits frontal areas but also relies on posterior parietal regions^65–68^.

By all accounts, the CE could be separated into distinct sub-functions that are performed by discrete cortical regions. Hence, the integration function was assumed to occur in the posterior parietal cortex and attention control was assumed to occur in the frontal cortex. These assumptions were supported by the notion that attention and information representations of WM were assigned to the frontal and parietal regions^69^. The two functions of the CE jointly control multisensory processing in WM. Specifically, the posterior parietal cortex is known to play a role in cognition functions^70, 71^, and this cortex can be considered a hub of multisensory information^44, 71–74^ that combines information on different sensory modalities to form a unified sensory space^75^. In particular, the inferior parietal lobule is a key location in the brain that is extensively connected with the auditory, visual, and somatosensory cortices. The neurons in this lobule are multimodal because they can process different types of stimuli simultaneously. This multisensory association of traits makes this lobule an ideal candidate for the integratinginformation from different modalities. Therefore, a﻿ccording to the classic theory of hierarchical processing,﻿﻿ it is possible that﻿ auditory and visual information of WM is initially processed within a specialized phonological loop and a visuospatial sketchpad^76^, and then these subcomponents of WM from different code systems are integrated in the posterior parietal cortex such as interior parietal lobule through the coordination ﻿of﻿ CE, thereby forming a unified multisensory presentation.

In addition, the results from the point-wise t-test and the scalp topography distributions of ERPs showed that frontal electrodes or areas participated in the integration effect at approximately 400 ms after stimuli onset. This finding may suggest that the frontal cortex exerts top-down attention control at a relatively later stage. This action of the frontal cortex could contribute to the monitoring﻿ of﻿ information integration because both the inferior frontal gyrus and the middle frontal gyrus involve in attention control^77, 78^. The modulation of inferior frontal gyrus excitability via rTMS induces a significant improvement in attentional task performance^79^, and the middle frontal gyrus is also engaged in a sustained mnemonic response in high-demand WM tasks^80^. The information integration appearing in the posterior parietal cortex is supervised via top-down attention control of the frontal cortices to facilitate retrieval in the future.

Early ERP studies in humans demonstrated that brain responses differ between paired multisensory stimuli and unisensory stimuli presented alone starting within the first 100 ms after stimulus onset^81–83^, and they showed that primary sensory cortices were potential source of these differences^84–86^. The early multisensory interactions are strongly related to the stimulus input characteristics and could reflect bottom-up processing because the presence of such interactions can be retained despite attentional suppression^87^. Thus, early multisensory interactions are a hallmark of bottom-up multisensory processes^43^ and may be automatic^88, 89^. However, these findings differed from the result of the present study﻿ that semantic integration of multisensory WM occured at a relatively later stage,  because multisensory integration involving semantic stimuli reflects the influence of top-down processing^90, 91^ and may happen in the relatively later. For instance, the first convergence of audiovisual speech (letters and sounds) has been found to occur at latencies of approximately 225 ms and present delays between 380 and 450 ms after stimulus onset^92^. This results is consistent with the classical view that the integration of senses occurs at a later stage of processing after the initial unisensory information has been processed^91^.

In summary, the information from WM subsystems could be integrated into unified memory traces during the encoding stage, thereby facilitating memory retrieval. The multisensory integration of WM involves the coordination of distributed brain networks. The integration effect of WM occurs in the posterior parietal cortex, and the frontal cortices subsequently exert attention control over the  information ﻿integration﻿.

## Methods

### Participants

Thirty-eight neurologically normal paid volunteers (all right handed; aged 20–24 years old, mean = 23.16 years old; 22 males and 16 females) who reported having normal hearing and normal or corrected to normal vision participated in this study. Two participants (males) were excluded from the analysis because of extensive oculomotor artefacts. This study was approved by the Ethics Committee of the Third Military Medical University, China. All participants provided written informed consent prior to the start of the experiment. All experimental methods were conducted in accordance with the ethical guidelines determined by the National Ministry of Health, Labour and Welfare and the Declaration of Helsinki (BMJ 1991; 302:1194).

### Stimuli

Visual stimuli were obtained from the standard set of outline drawing figures^93, 94^. The selected pictures contained an equivalent number of objects from different semantic categories (e.g., animals, tools, instruments, vehicles, etc.) and were divided equally among experimental conditions. The auditory stimuli consisted of verbalizations that corresponded to the visual stimuli. For example, the sound of a cat meowing was paired with the picture of a cat. All of the sound files were downloaded from a website (http://www.findsounds.com) and modified with audio editing software (Adobe Audition version 5.0) according to the following parameters: 16 bit; 44,100 Hz digitization; and 0.6 s sound duration with a 10 ms linear amplitude enveloping at sound onset and offset to avoid clicks.

In the present study, we only considered the semantic matching of AV stimuli relative to unisensory stimuli because prior studies (as mentioned in the Introduction) demonstrated that in many cases, a positive effect on subsequent memory recognition was only observed for semantically congruent and not incongruent AV presentations. Thus, three stimulus types were employed: auditory unimodal, visual unimodal, and congruent audiovisual (see appendix A). During the congruent AV condition, the recognition stimuli were only visual objects, which was similar to the condition in previous studies.

### Experimental procedures

Participants sat in a quiet and comfortable room and performed a delay-matched WM task during the three experimental blocks (A, V, and AV), which each contained 30 trials. The details of each trial in the three blocks are displayed in Fig. 1A. All three stimuli types were presented at the encoding stage for a duration of 0.6 s, which was followed by 2 s of delay, and then the probe stimulus appeared until the participant responded within the 3 s time limit. The visual stimuli were presented on a 17-inch computer monitor that subtended the visual angle by approximately 6.5 degrees and had a black background, and semantically related sounds were delivered binaurally at an intensity level of 70 dB via earphones. Congruent AV stimuli were simultaneously presented. The inter-trial interval ranged from 1500 to 3000 ms, and blocks were randomly varied between participants. The participants were asked to judge whether the probe stimulus was same or not with the stimuli presenting during the encoding stage with a key response (Yes and No responses corresponding to the "F" and "K" keys on the keyboard), and presented and unpresented probe stimuli were split in half. Breaks were encouraged between blocks to maintain high concentration and prevent fatigue. At the end of each block, the percentage of correct responses was displayed on the monitor. The stimulus delivery and behavioural responses recordings were controlled using E-prime 2.0 (Psychology Software Tools, Inc., Pittsburgh, PA; http://www.pstnet.com/eprime).

### EEG acquisition and preprocessing

A high-density electroencephalography (EEG) recording was acquired with a QuickAmp amplifier (Compumedics USA, El Paso, TX, USA) using 64 Ag/AgCl electrodes (extended 10–20 system). Horizontal and vertical electrooculograms were recorded with four bipolar electrodes placed on the outer canthus of each eye and above and below the right eye. Reference electrodes were placed on the bilateral mastoids. The electrode impedances were kept below 5 KΩ on average. The EEG activity was amplified using 0.01–100 Hz band-passed filters and sampled at 1000 Hz. EEG data were pre-processed with MATLAB 2013b (MathWorks, USA) and the EEGLAB13.4.4b toolbox^95^. EEG data at each electrode were re-referenced to the average of the left and right mastoids before further analysis. Then, the signal was digitally filtered off-line with a band-pass filter of 0.1–100 Hz. Time windows of 200 ms before and 600 ms after the onset of stimulus were segmented from the EEG (only focused on the encoding stage of WM). Epochs with amplitude values exceeding ± 100 uV at any electrode and those containing blinks, eye movements, or incorrect responses were rejected and excluded from the analysis. In addition, the datasets analyzed during the current study are available from the corresponding author on reasonable request.

## Data analysis

### Behavioural analysis

RTs were recorded on-line for the three types of trials. Only RT values associated with correct responses were considered for the data analysis. Accuracy rates were calculated as the percentage of correct responses (correct hit and correct rejection). Further statistical analyses were conducted using IBM SPSS software V22 (IBM Corp., Armonk, NY, USA).

### ERP analysis

Because of the paucity of prior knowledge on defined latencies and locations for the integration effect in multisensory WM, point-wise comparisons between the ERP_AV_ and ERP_A+V_ from each electrode and time point were performed to detect when and where the integration effect occuring by the MUET^96^. A cluster-based permutation test was used to control the family-wise error rate based on the statistical significance and the proximity of nearby time points and electrodes into clusters. This approach is probably the most powerful mass univariate procedure for the detection of broadly distributed effects^97^. The threshold of the test-wise alpha value for cluster inclusion was set at the 0.05. The number of permutations in the present test was 5000. Pairs of time values specified the beginning and end of a time window at approximately 0–600 ms.

### sLORETA analysis

Source localization was conducted to explore the originator in the integration effect of WM by SLORETA. sLORETA resolves the inverse problem by assuming synchronous and simultaneous activation of neighbouring neurons^98^. The localization accuracy of sLORETA has been validated by combining LORETA with tomographic techniques, such as EEG/fMRI^99^. In sLORETA, the intracerebral volume is partitioned in 6239 voxels at a 5 mm spatial resolution, and the standardized current density at each voxel is then calculated in a realistic head model. In the present study, the source location was investigated using voxel-wise randomization tests with 5000 permutations based on statistical non-parametric mapping^100^. Voxels with significant differences (corrected p-value < 0.01) were located in the MNI brain and Brodmann areas provided by the software.

### Statistics

A repeated measures analysis of variance was used to analyse the behavioural and ERP data. The condition (A, V, and AV) and electrode (only for ERP data) factors were used as within-subject factors. A Greehouse-Geisser correction was applied to adjust the degrees of freedom. When necessary, post hoc comparisons were performed with the Bonferroni correction. Kolmogorov-Smirnov tests indicated that all variables used for the analysis were normally distributed (all z < 0.8, p > 0.3).
